# Gut Microbiota Comparison Between Intestinal Contents and Mucosa in Mice With Repeated Stress-Related Diarrhea Provides Novel Insight

**DOI:** 10.3389/fmicb.2021.626691

**Published:** 2021-02-23

**Authors:** Chen-Yang Zhang, Xin-Xin Peng, Hao-Qing Shao, Xiao-Ya Li, Yi Wu, Zhou-Jin Tan

**Affiliations:** ^1^College of Traditional Chinese Medicine, Hunan University of Chinese Medicine, Changsha, China; ^2^Hunan Key Laboratory of Traditional Chinese Medicine (TCM) Prescription and Syndromes Translational Medicine, Changsha, China; ^3^Department of Pediatrics, The First Affiliated Hospital of Hunan University of Chinese Medicine, Changsha, China

**Keywords:** gut microbiota, intestinal content, intestinal mucosa, repeated stress-related diarrhea, functional bowel disorders, mice

## Abstract

Repeated stress-related diarrhea is a kind of functional bowel disorders (FBDs) that are mainly stemming from dysregulation of the microbiota–gut–brain axis mediated by a complex interplay of 5-hydroxytryptophan (5-HT). Intestinal content and intestinal mucosa microbiota belong to two different community systems, and the role of the two microbiota community systems in repeated stress-related diarrhea remains largely unknown. In order to ascertain the difference in composition and the potential function between intestinal content and intestinal mucosa microbiota response on repeated stress-related diarrhea, we collected intestinal contents and mucosa of mice with repeated stress-related diarrhea for 16S rRNA PacBio SMRT gene full-length sequencing, and with the digital modeling method of bacterial species abundance, the correlations among the two microbiota community systems and serum 5-HT concentration were analyzed. We found that the microbiotal composition differences both in intestinal contents and mucosa were consistent throughout all the phylogenetic ranks, with an increasing level of resolution. Compared with intestinal content microbiota, the diversity and composition of microbiota colonized in intestinal mucosa are more sensitive to repeated stress-related diarrhea. The PICRUSt2 of metagenomic function analysis found that repeated stress-related diarrhea is more likely to perturb the intestinal mucosa microbiota metagenomic functions involved in the neural response. We further found that the mucosal microbiota-based relative abundance model was more predictive on serum 5-HT concentration with the methods of machine-learning model established and multivariate dimensionality reduction (*R*^2^ = 0.876). These findings suggest that the intestinal mucosa microbiota might serve as a novel potential prediction model for the serum 5-HT concentration involvement in the repeated stress-related diarrhea, in addition to focusing on its mechanism in the gastrointestinal dysfunction.

## Background

Psychosocial factors are associated with functional bowel disorders (FBDs) and might influence symptom severity (Black et al., 2020; Jones et al., 2020). Diarrhea as a common symptom of FBDs has been linked to life stress (Greenland et al., 2016; Chan et al., 2019). Functional diarrhea is more common in people with psychological comorbidity than in the rest of the general population, for its stemming from dysregulation of the gut–brain axis which is mediated by a complex interplay of intestinal microbiota imbalance and central nervous system processing (Chan et al., 2019; Black et al., 2020). Consistent with our previous research (Zhang et al., 2020a), many studies suggest that the tryptophan metabolic pathway plays an important role in the microbiota–gut–brain axis, synchronizes the gut with the central nervous system, and modifies the psychosocial stress (Doifode et al., 2020; Ma et al., 2020). Tryptophan metabolism disorders in intestinal microbiota cause 5-hydroxytryptophan (5-HT), an intermediate product of tryptophan metabolism, to activate the brain–gut nerve response and induce diarrhea (Spencer and Hu, 2020; Ming et al., 2021). FBDs associated with psychosocial factors present microbial changes with plausible relation with deficiency of 5-HT metabolism (Settanni et al., 2021). 5-HT, as an important signaling molecule in the gut targeting enterocytes, smooth muscles, and enteric neurons, activates both intrinsic and extrinsic primary afferent neurons to respectively initiate peristaltic influence many important processes including mood and gut homeostasis (Chandramowlishwaran et al., 2020). So, the content of 5-HT in serum can represent the intestinal microbiota participating in the brain–gut nerve response of stress-related diarrhea to a certain extent. Non-pharmacologic interventions such as stress and mental health counseling were useful, but it is difficult to popularize it in functional diarrhea patients (Ma et al., 2021), and the development of specific drugs is necessary. The research on the mechanism of psychological factors related to diarrhea is conducive to the development of drug therapy.

Although many studies recognized that intestinal content and intestinal mucosal microbiota belong to different community systems (Gao et al., 2018; Maharshak et al., 2018; Vasapolli et al., 2019; Lin et al., 2020), the role of the two microbiota community systems in disease remains largely unknown. Studies of gut microbiota mainly focus on the diversity and composition of fecal or content microbiome (Wang X. et al., 2020), but the fecal or content microbiome is not representative of the mucosal microbiome (Vasapolli et al., 2019). The difference of fecal and mucosal microbiome is not just in diversity and composition. The study found that two separate microbial populations, the lumen-associated and the mucosa-associated microbiota, responded to different environmental factors (Borgo et al., 2018). What is more, clinical studies have found that the efficacy of fecal microbiota transplantation (FMT) in treating gastrointestinal diseases varies greatly among individuals, and the mucosal microbiome can provide diagnosis and treatment strategies (Sarrabayrouse et al., 2020; Sokol et al., 2020). Therefore, research on the microecological mechanism of functional mental stress-related diarrhea, with the pathological basis of extensive and mild intestinal inflammation, should pay more attention to the differences between intestinal content and intestinal mucosa microbiome.

In order to study the difference of composition and function between intestinal content and intestinal mucosal microbiota response on functional mental stress-related diarrhea, referring to our previous researches (Yuan et al., 2020; Zhang et al., 2020b), a model of repeated stress-related diarrhea was constructed by lavage with *Folium senna* extract combined with restraint and tail pinch stress in this study. The 16S rRNA PacBio Single Molecule, Real-Time (SMRT) gene full-length sequencing of intestinal content and mucosal microbiota was used to compare the differences in microbiota diversity, composition, and key bacteria. The differences of microbiota metagenomic function prediction in intestinal content and mucosa microbiota of the repeated stress-related diarrhea mice were further analyzed. Moreover, in our study, a machine-learning method of microbial relative abundance was used to establish a model to predict the serum 5-HT content in mice, and the correlation between the two microbiota community systems and 5-HT was comparatively analyzed to further explore the functional differences of intestinal content and mucosa microbiota on psychological stress-related diarrhea.

## Materials and Methods

### Animals

Kunming (wild type; WT) mice (10 week-old male, 20 ± 2 g on average) were purchased from the Hunan Slaccas Jingda Laboratory (SJA) Animal Company (Hunan, China), with license number SCXK (Xiang) 2016-0002. Kunming mice were raised at a shielded environment at the Animal Experiment Center of the Hunan University of Chinese Medicine with license number SYXK (Xiang) 2015-0003, in a 12/12 dark–light cycle (21 ± 2°C with a relatively constant humidity of 45 ± 10%), with *ad libitum* access to food and water. The experimental protocols used on animals were approved by the Animal Ethics and Welfare Committee of Hunan University of Chinese Medicine.

### Diarrhea and Repeated Stress Modeling

Ten mice were randomly divided into normal control group (c group) and repeated stress-related diarrhea model group (ds group). According to our previous studies (Yuan et al., 2020; Zhang et al., 2020b), the mice in the ds group were treated with *Folium senna* extract combined with restraint and tail pinch to induce diarrhea and repeated stress for 7 days continuously. Meanwhile, mice in the c group were given the same dose of normal saline. During the modeling period, the animal weight was recorded once a day.

### Detection of 5-HT With Enzyme-Linked Immunosorbent

The blood samples collected from the mandibular venous plexus were shaken in EDTA-K2 anticoagulant tube. After 20 min of standing, the blood samples were centrifuged at 4°C at 3,000 rpm for 15 min and the serum was collected by centrifugation (stored in a refrigerator at 4°C). According to the instructions of the kit, ELISA was used to detect the content of 5-HT in serum (Zhu et al., 2020).

### Intestinal Contents and Mucosa Collection

The intestinal tract tissue from the pylorus of the stomach to the ileocecus was cut longitudinally with sterile scissors to peel off the contents. Intestinal content samples in the normal control group (Cc group) and repeated stress-related diarrhea model group (Cds group) were collected in sterilized tubes and stored in a −80°C freezer for 16S rRNA PacBio SMRT gene full-length sequencing.

The mucosa of intestinal tract tissue stripped of contents was scraped with a slide. Intestinal mucosa samples in the normal control group (Mc group) and repeated stress-related diarrhea model group (Mds group) were collected in sterilized tubes and stored in a −80°C freezer for 16S rRNA PacBio SMRT gene full-length sequencing.

### 16S rRNA PacBio SMRT Gene Full-Length Sequencing

16S rRNA gene sequencing, PacBio SMRT sequencing technology, was used to accurately obtain the information of rRNA gene full-length sequence (Chen et al., 2020; Escapa et al., 2020). Total microbial genomic DNA of intestinal mucosa samples were extracted following the manufacturer’s instructions and stored at −20°C. Total microbial genomic DNA samples were extracted using the OMEGA DNA isolation kit (Omega, D5625-01, United States) following the manufacturer’s instructions. DNA concentration was determined by NanoDrop ND-1000 spectrophotometer (Thermo Fisher Scientific, Waltham, MA, United States). PCR amplification of the nearly full-length bacterial 16S rRNA genes was performed using the forward primer 27F (5′-AGAGTTTGATCMTGGCTCAG-3′) and the reverse primer 1492R (5′-ACCTTGTTACGACTT-3′). The extracted DNA was amplified with two-step PCR, with sample-specific 16 bp barcodes incorporated into the forward and reverse primers for multiplex sequencing in the second PCR step. Next, the TruSeq Nano DNA LT Library Prep Kit was used to prepare the sequencing library. The library was tested by Agilent high sensitivity DNA kit on the Agilent Bioanalyzer. Finally, the amplified DNA fragment was sequenced by MiSeq sequencer with 2 × 300 bp double end. The sequencing reagent was MiSeq reagent kit V3 (600 cycles). SMRT sequencing technology was performed using the PacBio Sequel platform at Shanghai Personal Biotechnology Co., Ltd (Shanghai, China).

### Bioinformatics and Statistical Analysis

All fastq files were quality controlled using QIIME 1.9.1 workflow. The remaining high-quality sequences were clustered into operational taxonomic units (OTUs) at 97% sequence identity with UCLUST (Edgar, 2010). The Biological Observation Matrix (BIOM) file was used for the downstream analysis with QIIME 1.9.1 pipeline and R language (v3.2.0). Alpha diversity was measured using the number of observed OTUs and Chao1, Simpson, ACE, and Shannon indices were rarified at the same sequencing depth, to present the observed species and diversity of the gut microbiota. Beta diversity analysis was performed to investigate the structural variation of microbial communities across samples using UniFrac distance metrics and principal coordinate analysis (PCoA). Composition of bacterial species and the relative abundance of KEGG orthologous groups (KOs) were plotted using the “UpsetView plot,” “CPCoA plot,” and “Pretty Heatmap” sections of Ehbio Cloud Platform^1^. The differences of bacterial relative abundance were calculated and plotted by Majorbio cloud platform^2^; the Wilcoxon rank sum test was used to analyze differences in the relative abundance of the microbiota. Linear discriminant analysis effect size (LEfSe) was performed to identify differences in the microbial structure between groups, with the default parameter^3^. PICRUSt2^4^ was used to identify differences in the metabolic pathways between each group against the KEGG (Douglas et al., 2020). ECharts software was used for the microbiota metagenomic function analysis and plotting^5^. DNA sequences of OTUs with statistical difference in relative abundance were used to dock with the KEGG Orthology (KO) system. Approximate distributions of differential community functional classifications (pathway and brite functional hierarchy) of the microbiota were obtained, assessing potential functional distribution information, and the four tiers of brite features were analyzed statistically in further detail. Prediction of LDA (linear discriminant analysis) algorithm analysis was performed by using the “MASS” package of R. The relative abundances of taxonomic bacterial species were merged and used for training and validation of a LDA linear judgment model. LDA can be used not only to solve the classification problem but also to reduce the dimension of data. Linear regression analysis was used to determine the quantitative relationship between serum 5-HT concentration and LDA microbiota relative abundance models.

Experiment results were presented as mean ± SEM. Bar plots and statistical analysis were generated using GraphPad Prism 7 by using one-way ANOVA and *t*-test. *P* < 0.05 was considered significant. Representation of the *P*-value is as follows: ^∗^*P* < 0.05, ^∗∗^*P* < 0.01, ^∗∗∗^*P* < 0.001, and N.S., not significant (*P* > 0.05).

## Results

### The Diversity and Composition of Microbiota Colonized in Intestinal Mucosa Are More Sensitive to Repeated Stress-Related Diarrhea

#### Bacterial DNA Sequences Are Divided by OTUs

In an attempt to evaluate the effect of repeated stress-related diarrhea on intestinal microbiota, 10 week-old Kunming WT mice were treated with *Folium senna* extract combined with restraint and tail pinch for 7 days to induce repeated stress-related diarrhea model. After modeling, samples were harvested for sequencing. Twenty samples (10 intestinal contents and 10 intestinal mucosa) were collected and sequenced in 16S rRNA single molecule sequencing, generating 190,874 high-quality, ∼1,500 bp paired-end reads, with average 9,543 reads for each sample. Reading length of full gene sequencing was concentrated at 1,500 bp (Supplementary Figure S1A). The species accumulation curve showed that OTUs reached asymptote with the increase of sampling (Supplementary Figure S1B). The number of OTUs can be compared in different samples under the same sequencing depth in the sparse curve, so as to measure the diversity of each sample to a certain extent (Supplementary Figure S1C). The Venn of OTU number showed that there were differences in the composition of intestinal contents and mucosa (Supplementary Figure S1D).

#### Microbiota Diversity Analysis and OTU Taxonomic Status Identification From Phylum to Genus

**FIGURE 1:**
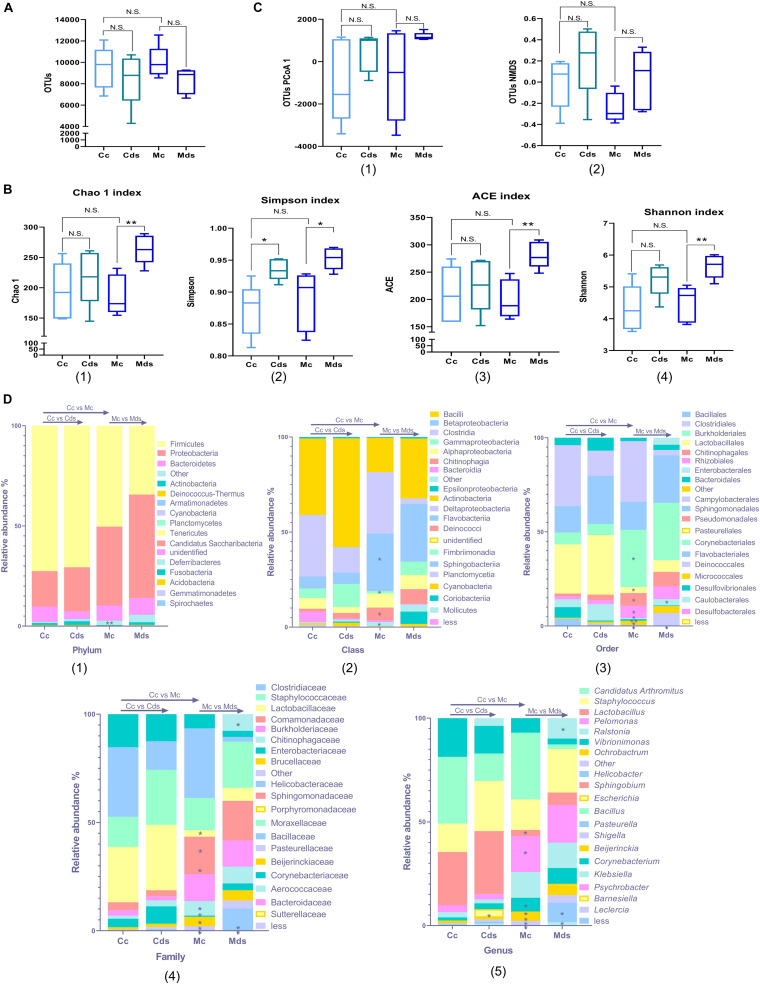
Repeated stress-related diarrhea alters intestinal microbiota diversity and composition. **(A)** Box plots of observed operational taxonomic unit (OTUs). **(B)** Box plots of α-diversity index of OTUs (Chao1, Simpson, ACE, and Shannon). **(C)** Box plots of β-diversity index of PCoA1 and NMDS of OTUs, represented by the space distance among groups. **(A–C)** *P < 0.05, **P < 0.01; N.S., not significant (*P* > 0.05). **(D)** Relative mean abundance of (1) phyla, (2) class, (3) order, (4) family, and (5) genus in the intestinal contents and mucosa; *P < 0.05, **P < 0.01, Wilcoxon rank sum test. Cc, intestinal contents in control mice; Cds, intestinal contents in repeated stress-related diarrhea mice; Mc, intestinal mucosa in control mice; Mds, intestinal mucosa in repeated stress-related diarrhea mice.

The results showed that there were no significant differences in OTU number and diversity in the contents and mucosa of normal mice (Figures 1A–C). Diversity analysis showed that the repeated stress-related diarrhea did not change the OTU number of intestinal bacteria in the contents and mucosa (Figure 1A), while it significantly perturbed the alpha diversity of mucosal microbiota, represented by Chao1 (*P* = 0.0031), Simpson (*P* = 0.0184), ACE (*P* = 0.0029), and Shannon (*P* = 0.0049) indices (Figure 1B). There was no significant difference in the beta diversity of OTUs represented by PCoA and non-metric multidimensional scaling (NMDS) (Figure 1C and Supplementary Figure S2).

The bacterial communities were analyzed systematically over the taxonomic ranks from phylum to phylotype. With the Wilcoxon rank sum test, we compared the effects of repeated stress-related diarrhea on intestinal content and intestinal mucosa microbiota in mice and the differences of intestinal content and intestinal mucosal microbiota in normal mice. The repeated stress-related diarrhea did not alter the composition of intestinal microbiota at the ranks of both phylum and class, but there was a significant difference between intestinal contents and intestinal mucosa in normal mice. The differences were evident at the phylum level between intestinal contents and intestinal mucosa; members of the phylum Firmicutes accounted for roughly 50% of the total bacterial community, and in intestinal mucosa, the relative abundance of phylum Firmicutes decreased, but not statistically significant (Figure 1D1). At the phylum level, there were significant differences in the changes of rare bacteria without classification (represented as “other” in Figure 1D1, *P* = 0.0079). At the class level, the intestinal mucosa was characterized by a low abundance of Betaproteobacteria (*P* = 0.0317), Chitinophagia (*P* = 0.0159), and rare bacteria (*P* = 0.0119) and a high abundance of Gammaproteobacteria (*P* = 0.0317) (Figure 1D2). These differences were consistent throughout all the phylogenetic ranks, with an increasing level of resolution (Figure 1D).

It was not until the level of order classification that we found the difference of intestinal microbiota composition, especially the intestinal mucosa microbiota. At the order level, Myxococcales (*P* = 0.0231) disappeared in the intestinal mucosa corresponding to an exhaustion of the family Archangiaceae and genus *Stigmatella*, and Enterobacterales (*P* = 0.0465) showed a higher abundance in the intestinal mucosa of mice with repeated stress-related diarrhea (Figures 1D3–5). The family Bacillaceae (corresponding to the genus *Bacillus*) was present in the intestinal mucosa of normal mice only at a negligible abundance, but showed a significantly higher abundance in the intestinal mucosa of mice with repeated stress-related diarrhea (*P* = 0.0449) (Figures 1D4, D5). After the intervention of modeling factors, the relative abundance of family Peptococcaceae (corresponding to the genus *Klebsiella*) in intestinal mucosa decreased significantly (*P* = 0.0209) (Figures 1D4, D5). Meanwhile, in the intestinal contents of mice with repeated stress-related diarrhea, only the genus *Escherichia* showed significant proliferation (*P* = 0.0465), which indicated that repeated stress-related diarrhea had less effect on the intestinal contents than on the intestinal mucosa microbiota (Figure 1D5).

#### Analysis of Microbiota Species Rank Composition

**FIGURE 2:**
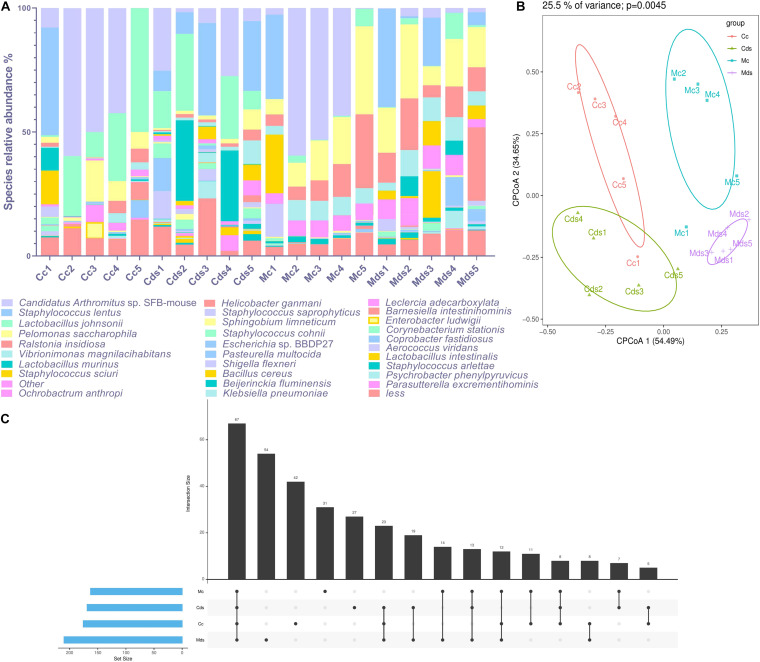
Repeated stress-related diarrhea significantly perturbs the diversity and composition of intestinal microbiota at the species level. **(A)** The composition of dominant taxonomic species in each sample. **(B)** Cluster analysis of PCoA based on taxonomic bacterial species. **(C)** UpsetView of taxonomic bacterial species in groups. Cc, intestinal contents in control mice; Cds, intestinal contents in repeated stress-related diarrhea mice; Mc, intestinal mucosa in control mice; Mds, intestinal mucosa in repeated stress-related diarrhea mice.

*Candidatus Arthromitus* sp. SFB-mouse colonized the most in the intestinal contents (22.56%), while *Pelomonas saccharophila* colonized the most in intestinal mucosa (17.72%) in the current study. The composition of intestinal content microbiota is different from that of intestinal mucosa; as the dominant species (top 30 abundance), *Helicobacter ganmani* accounts for 3.02% of intestinal mucosa microbiota, but only 0.30% of intestinal contents (Figure 2A). Although the above results show that there was no significant difference in the beta diversity of OTUs represented by PCoA and NMDS (Figure 1C and Supplementary Figure S2), taxonomic analysis identified by the OTUs of microbiota showed a significant difference at the species level. There were significant differences in the composition of bacterial species within intestinal content and mucosal microbiota (*P* = 0.0045; principal components 1 and 2 of cluster analysis contained 89.14% data information), and obvious cluster separation was shown in the PCoA diagram (Figure 2B). There was a difference in the composition of bacterial species both in the contents and mucosa of repeated stress-related diarrhea mice (Figure 2C); especially in the mucosa, the number of specific bacterial species was the highest (Figure 2C). Therefore, our study is based on the abundance of taxonomic species rank, rather than the OTUs, to analyze the characteristics of intestinal content and mucosal microbiota affected by repeated stress-related diarrhea.

### Identification of Key Bacteria Associated With Repeated Stress-Related Diarrhea in the Intestinal Contents and Mucosa

**FIGURE 3:**
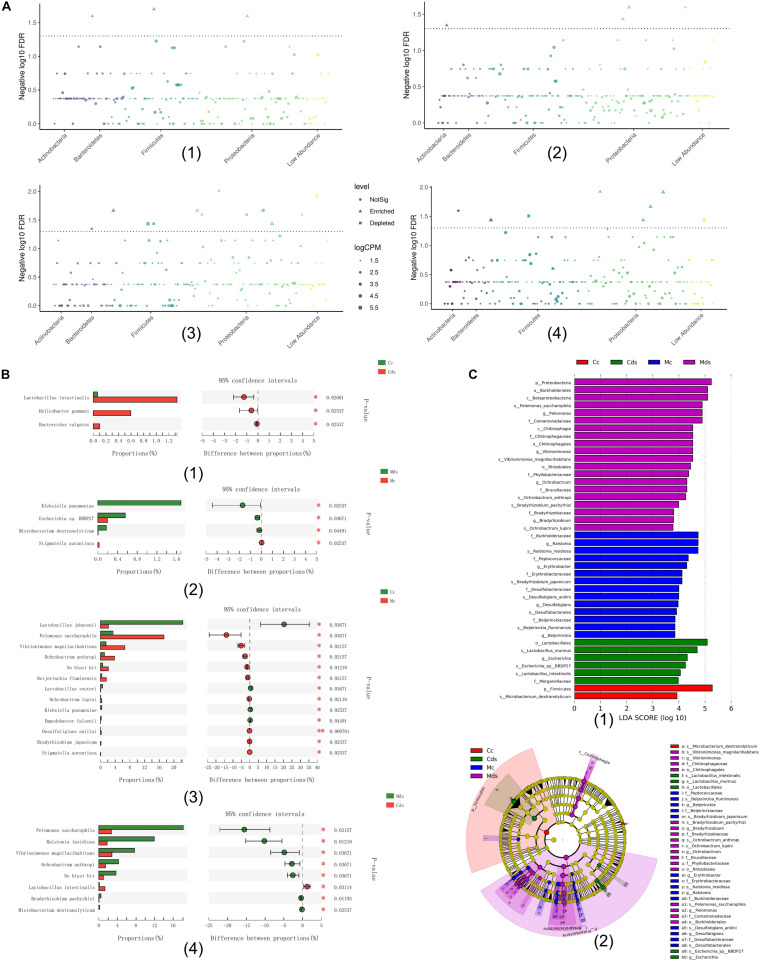
The key bacteria associated with repeated stress-related diarrhea in the intestinal contents and mucosa were identified. **(A)** Classification and significance of target taxonomic bacterial species at the phylum level; the element represents each bacterial species, the element size represents the relative abundance, and the element color represents the taxon level. (1) Comparison of intestinal content taxonomic bacterial species between repeated stress-related diarrhea mice and normal mice; (2) comparison of intestinal mucosa taxonomic bacterial species between repeated stress-related diarrhea mice and normal mice; (3) comparison of taxonomic bacterial species between intestinal contents and intestinal mucosa in control mice; (4) comparison of taxonomic bacterial species between intestinal contents and intestinal mucosa in repeated stress-related diarrhea mice. **(B)** Bacterial relative abundance significantly changed in the Wilcoxon rank sum test with *P* < 0.05. **(C)** (1) Cladogram generated from the LEfSe analysis indicating the phylogenetic distribution from phylum to species of the microbiota. (2) Histogram of LDA scores to identify differentially abundant bacterial species (LDA score ≥ 2, *P* < 0.05). *P < 0.05, **P < 0.01. Cc, intestinal contents in control mice; Cds, intestinal contents in repeated stress-related diarrhea mice; Mc, intestinal mucosa in control mice; Mds, intestinal mucosa in repeated stress-related diarrhea mice.

Comparing the relative abundance at the species level, among the four groups, the Wilcoxon rank sum test (*P* < 0.05) showed that repeated stress-related diarrhea significantly altered the microbiotal composition both in intestinal contents and mucosa (Figures 3A,B). Among the four groups, bacterial species with significant difference in relative abundance mostly belonged to Proteobacteria (Figure 3A), and repeated stress-related diarrhea resulted in 28 bacterial relative abundance to be significantly changed (Wilcoxon rank sum test with *P* < 0.05) (Figure 3B).

Moreover, LEfSe analysis (Li et al., 2020) identified 42 signature bacterial taxa that were differentially altered, with an LDA score > 2, showing that there was a significant structural difference among the four groups (Figure 3C), in which 13 bacterial species were identified as key discriminants. All these results demonstrated that repeated stress-related diarrhea may be a key modulator of the microbiota composition, and the key bacteria may be able to identify repeated stress-related diarrhea disease.

### Repeated Stress-Related Diarrhea Is More Likely to Perturb the Microbiota Metagenomic Potential Function in the Intestinal Mucosa

#### Microbiota Metagenomic Function Prediction Based on the Pathway

**FIGURE 4:**
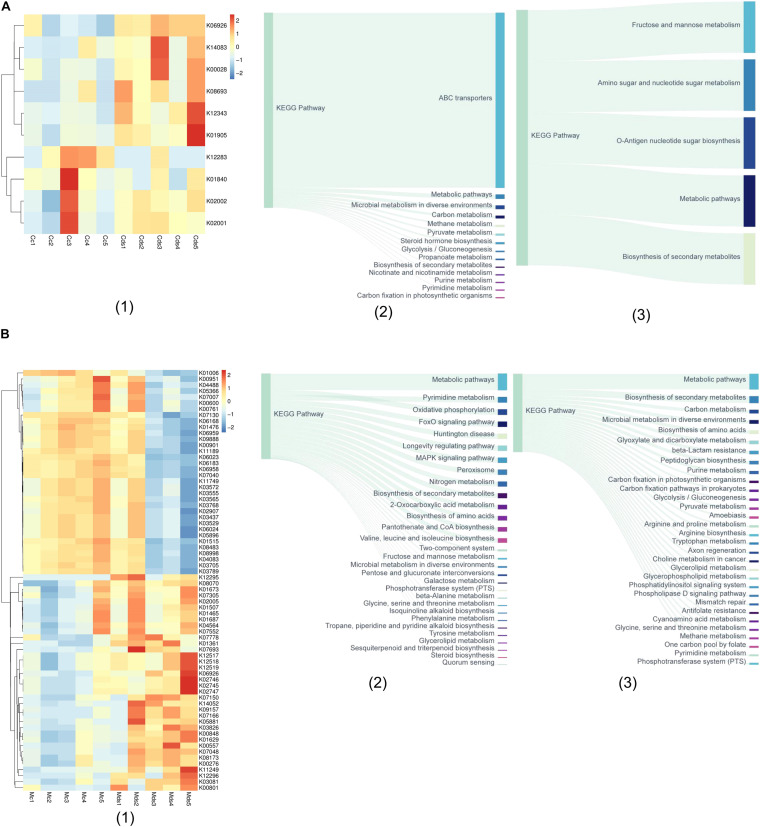
The metagenomic function prediction of the pathway based on the KEGG database. **(A)** The main KEGG pathway analysis of bacterial genes in intestinal contents of repeated stress-related diarrhea mice. (1) The relative abundance of differentially expressed KOs in intestinal content microbiota of repeated stress-related diarrhea mice; (2) the main KEGG pathway of the KOs with significantly increased expression in intestinal contents of repeated stress-related diarrhea mice; (3) the main KEGG pathway of the KOs with significantly decreased expression in intestinal contents of repeated stress-related diarrhea mice. **(B)** KEGG pathway analysis of bacterial genes in intestinal mucosa of repeated stress-related diarrhea mice. (1) The relative abundance of differentially expressed KOs in intestinal mucosa microbiota of repeated stress-related diarrhea mice; (2) the main KEGG pathway of the KOs with significantly increased expression in intestinal mucosa of repeated stress-related diarrhea mice; (3) the main KEGG pathway of the KOs with significantly decreased expression in intestinal mucosa of repeated stress-related diarrhea mice. Cc, intestinal contents in control mice; Cds, intestinal contents in repeated stress-related diarrhea mice; Mc, intestinal mucosa in control mice; Mds, intestinal mucosa in repeated stress-related diarrhea mice.

With the PICRUSt2 of metagenomic function prediction to assess the microbiota functional difference between the intestinal contents and mucosa (Douglas et al., 2020; Wang Z. et al., 2020), it was observed that repeated stress-related diarrhea is more likely to be altered of the microbiota function in the intestinal mucosa, represented by the analysis against the KEGG database. The microbiota composition data were “mapped” to the Greengenes database and against the KEGG database, and the sequencing of 20 samples resulted in 5,734 KOs. We found that repeated stress-related diarrhea resulted in the relative abundance of 10 KOs (with the average of 2,840 OTUs for each sample) in the intestinal contents (Figure 4A1) and in the relative abundance of 70 KOs (with the average of 90,500 OTUs for each sample) in the intestinal mucosa (Figure 4B1) to be significantly changed (Wilcoxon rank sum test with *P* < 0.05).

In the intestinal contents, repeated stress-related diarrhea resulted in a significant increase in the relative abundance of KOs in the ABC transporter pathway (Figure 4A2), and the relative abundance of KOs was significantly reduced in metabolic pathways, mainly including the biosynthesis and metabolism of fructose, mannose, amino sugar, nucleotide sugar, etc. (Figure 4A3). The metagenomic functional analysis found that, in the intestinal contents, the repeated stress-related diarrhea mainly reduced the abundance of bacteria with the function of carbohydrate metabolism.

Metagenomic functional analysis of intestinal mucosa sequencing, based on the 36 KOs with significantly increased relative abundance, showed that pyrimidine metabolism, oxidative phosphorylation, FoxO signaling pathway, and MAPK signaling pathway were higher in the taxa present in the intestinal mucosa of mice with repeated stress-related diarrhea compared with the control group (Figure 4B2). The 34 KOs with significantly decreased relative abundance in the intestinal mucosa indicated that carbon metabolism, biosynthesis of amino acids, and glyoxylate and dicarboxylate metabolism were moderately altered in response to repeated stress-related diarrhea (Figure 4B3). It can be seen that repeated stress-related diarrhea has a wide range of effects on the potential function of intestinal mucosa microbiota, mainly for the increased abundance of flora involved in immunity and inflammation and the decreased abundance of flora related to digestion.

#### The Microbiota Metagenomic Function Prediction Based on Brite Functional Hierarchy

**FIGURE 5:**
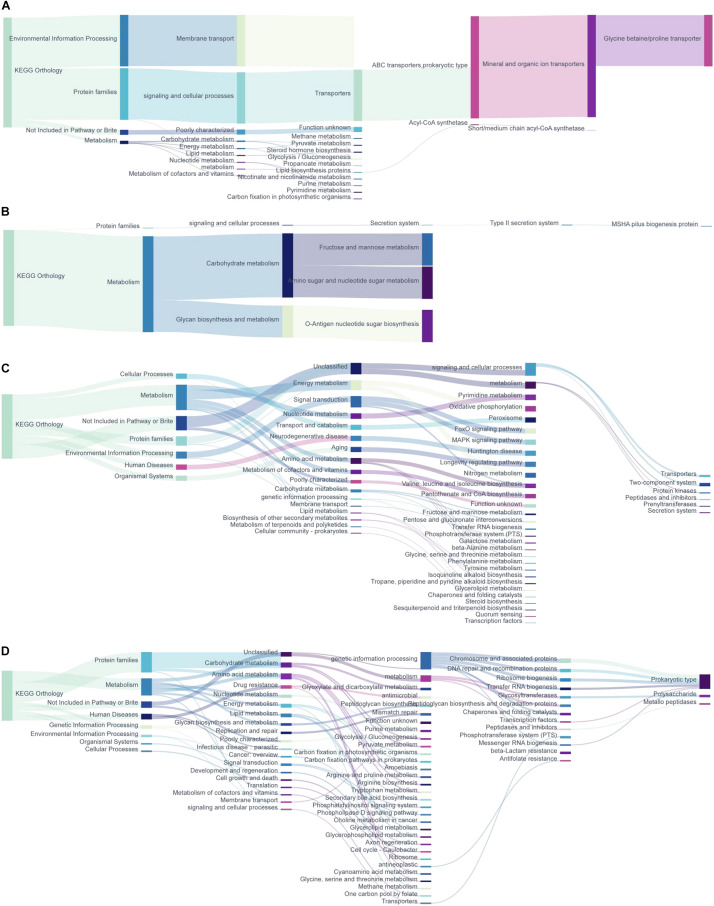
The metagenomic function prediction of the KEGG Orthology. **(A)** KEGG Orthology of the KOs with significantly increased expression in intestinal contents of repeated stress-related diarrhea mice. **(B)** KEGG pathway of the KOs with significantly decreased expression in intestinal contents of repeated stress-related diarrhea mice. **(C)** KEGG Orthology of the KOs with significantly increased expression in intestinal mucosa of repeated stress-related diarrhea mice. **(D)** KEGG Orthology of the KOs with significantly decreased expression in intestinal mucosa of repeated stress-related diarrhea mice.

According to the annotation information of KOs with significantly changed relative abundance discussed above, we further analyzed the functional mechanism of the intestinal microbiota (Figure 5). The abundance of ABC transporter function in prokaryotes increased in the intestinal contents of repeated stress-related diarrhea mice, which mainly affected the bacterial glycine betaine/proline metabolism (Figure 5A). Repeated stress-related diarrhea reduced the bacterial abundance with the function of carbohydrate metabolism in the intestinal contents (Figure 5B). Repeated stress-related diarrhea changed widely the metagenomic function of bacteria in the intestinal mucosa including metabolism, genetic information processing, environmental information processing, cellular processes, organismal systems, and human diseases. In the intestinal mucosa, bacteria related to energy metabolism including nitrogen metabolism and oxidative phosphorylation increased in repeated stress-related diarrhea mice; in the amino acid metabolic pathway, repeated stress-related diarrhea increased the gene expression of valine, leucine, and isoleucine biosynthesis; moreover, in repeated stress-related diarrhea mice, the intestinal microbiota may affect nervous system diseases through FoxO signaling pathway and MAPK signaling pathway (Figure 5C). The main gene functions of intestinal mucosal microflora decreasing significantly in mice were prokaryote genetic information processing (i.e., chromosome and associated proteins, etc.), carbohydrate metabolism (i.e., glyoxylate metabolism, dicarboxylate metabolism, glycolysis/gluconeogenesis, etc.), and amino acid metabolism (i.e., arginine, proline, and tryptophan metabolism) in repeated stress-related diarrhea (Figure 5D).

### Relative Abundance of Mucosal Microbiota-Based Machine-Learning Model Was Established

**FIGURE 6:**
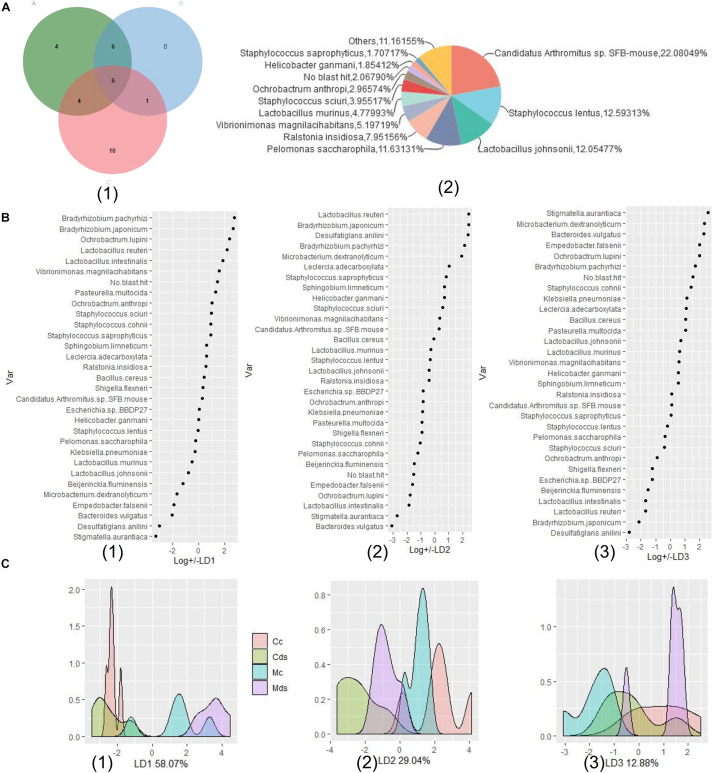
Relative abundance of microbiota-based machine-learning model based on LDA (linear discriminant analysis). **(A)** Identification of the signature gut microbiota, associated with repeated stress-related diarrhea. (1) To detect signature bacterial markers in response to the repeated stress-related diarrhea, we performed comparison of differences between groups (in green A), LEfSe analysis (in blue B), and relative abundance analysis (in red C) to identify the 31 key bacterial species in response to repeated stress-related diarrhea in the discovery phase. (2) The proportion of relative abundance of the 31 key bacterial species. **(B)** The relevance of each bacteria in the predictive model was assessed using learning LDA machine-learning classifier in the relative abundance of the 31 key bacterial species, and there are three LDA models, LD1 (1), LD2 (2), and LD3 (3), which can make good classification according to the relative abundance of bacteria. **(C)** A mathematical model based on the 31 key bacterial species was used to predict the sampling group. (1) Density distribution of samples on the LD1 model (58.07%); (2) density distribution of samples on the LD2 model (29.04%); (3) density distribution of samples on the LD3 model (12.88%).

In order to understand the correlation between the composition of intestinal microbiota and repeated stress-related diarrhea, we modeled the composition and structure of intestinal microbiota according to the relative abundance of bacterial species and conducted multivariate dimensionality reduction. LDA linear discriminant analysis is a classical dimension reduction method, and it is also a supervised machine-learning dimension reduction technology. According to the requirements of the LDA method for data type, we selected the key bacteria for modeling. Referring to the above analysis results, comparison of differences between groups, LEfSe analysis, and relative abundance analysis identified the 31 key bacterial species in response to repeated stress-related diarrhea (Figure 6A). The relative abundance of taxonomic bacterial species were merged and used for training and validation of a LDA machine-learning classifier. We constructed a prediction model based on learning LDA machine-learning classifier in the relative abundance of the 31 key bacterial species, which could help us select the best independent parameter. Using LDA, we identified the parameter of key bacterial species to build a prediction model for each environmental factor such as microbiota of repeated stress-related diarrhea, intestinal contents, and intestinal mucosa (Figure 6B). The relative abundances of the 31 key bacterial species were processed including dimensionality reduction to LD1, LD2, and LD3, which contain 58.07, 29.04, and 12.88% of the data information, respectively (Figure 6C). The LDA prediction models LD1, LD2, and LD3 can accurately distinguish which environmental factors the samples belong to Figure 6C, and the prediction accuracy of the model is 100%.

### Relative Abundance of Mucosal Microbiota Was More Predictive on Serum 5-HT Concentration

**FIGURE 7:**
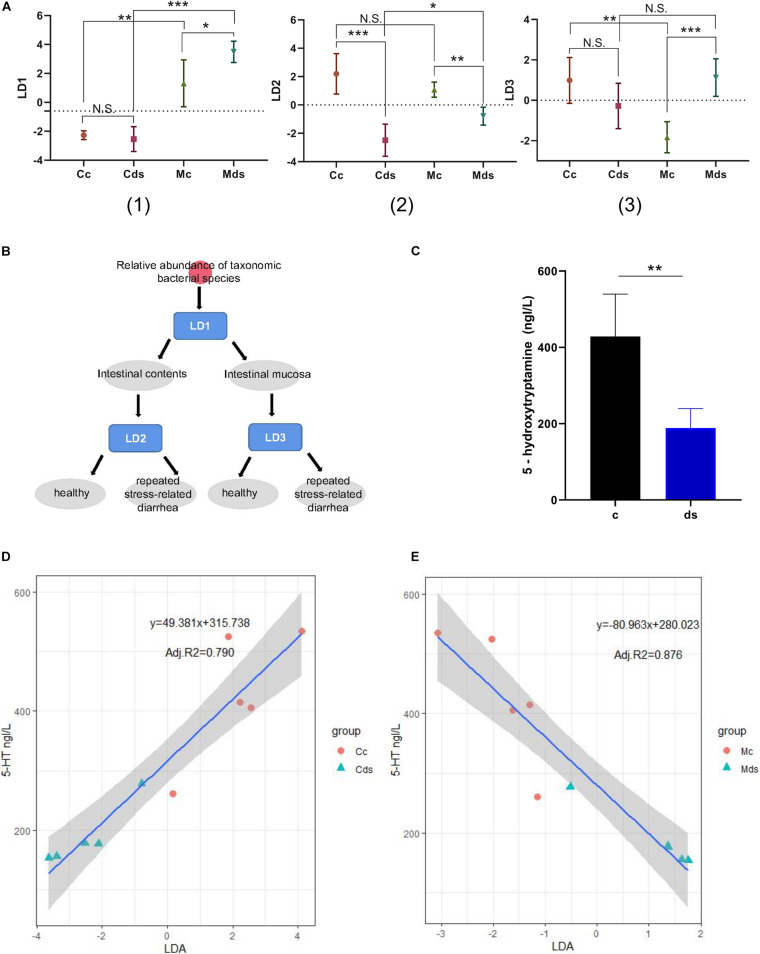
Establishment of a prediction model of 5-HT concentration based on bacterial relative abundance. **(A)** Prediction and classification of samples by the relative abundance of microbiota-based machine-learning models LD1 (1), LD2 (2), and LD3 (3). **(B)** Schematic diagram of the bacterial relative abundance model discrimination. **(C)** Serum 5-HT concentration. **(D)** Correlation between serum 5-HT concentration and intestinal content microbiota. **(E)** Correlation between serum 5-HT concentration and intestinal mucosa microbiota. *P < 0.05, **P < 0.01, ***P < 0.001; N.S., not significant (*P* > 0.05). Cc, intestinal contents in control mice; Cds, intestinal contents in repeated stress-related diarrhea mice; Mc, intestinal mucosa in control mice; Mds, intestinal mucosa in repeated stress-related diarrhea mice.

After the LDA discriminant modeling analysis, we found that the discriminant model LD1 has a good classification function for intestinal content and intestinal mucosal microbiota; model LD2 can better distinguish whether the intestinal content microbiota comes from healthy mice or from repeated stress-related diarrhea mice; and the discriminant model LD3 has a good classification function for the intestinal mucosal microbiota of healthy mice and diarrhea mice (Figures 7A,B). Moreover, the comprehensive intervention modeling led to the low expression of 5-HT (*P* = 0.0023) in diarrhea and repeated stress mice (ds group), compared with control mice in the c group (Figure 7C). 5-HT is a key inhibitory neurotransmitter for bacterial community to participate in the interaction of brain–gut nerves. We speculated whether the relative abundance of intestinal microbiota could be used as a biomarker to predict serum 5-HT. Linear regression analysis is a statistical analysis method that uses regression analysis in mathematical statistics to determine the quantitative relationship between two or more variables, and we established a linear regression model to predict serum 5-HT concentration in mice. On the LDA models, we built a prediction model for each environmental factor. We found that the LD2 model could predict serum 5-HT concentration by the relative abundance of intestinal content microbiota (*P* = 0.0003), and the LD3 model could predict serum 5-HT content by relative abundance of intestinal mucosal microbiota (*P* < 0.0001). Utilizing these models to predict the serum 5-HT concentration resulted in adjusted *R*^2^ of 79.0% in the microbial abundance model of intestinal contents (Figure 7D) and 87.6% in the microbial abundance model of intestinal mucosa (Figure 7E). Notably, the mucosal microbiota-based prediction model for the serum 5-HT concentration was predicted with higher confidence.

## Discussion

Here, based on 16S rRNA PacBio SMRT gene full-length sequencing, we provide a comprehensive description of the biodiversity and taxonomic bacterial species composition of microbial communities of two regions (intestinal contents and intestinal mucosa), which respond to repeated stress-related diarrhea. The novel finding of our study is that repeated stress-related diarrhea is more likely to perturb the biodiversity, composition, and function of microbiota in the intestinal mucosa compared with the microbiota in the intestinal contents. As recognized in a recent study, the microbiota colonized in different parts of the human gastrointestinal tract has its own specific community structure, and the different microbiota formed an interrelated and relatively independent microbiological system (Vasapolli et al., 2019). In healthy people, the intestinal mucosa is the site of continual interactions between microbes, tissue cells, and the immune system (Seo et al., 2020). A latest finding was reported that mucosal-associated invariant T (MAIT) cells interact with local microbiota and integrate multiple signals, so it plays an important role in local inflammation (Ioannidis et al., 2020). The microbiome at homeostasis colonized in intestinal mucosa can antagonize pathogenic bacteria, clear endotoxin, and play a role of resistance to external environmental factors (Seo et al., 2020); in turn, the intestinal epithelial cells can also affect the community structure of intestinal mucosal microbiota (Yoo et al., 2020). These researches all explain why intestinal mucosal microbiota in this study is highly susceptible to repeated stress-related diarrhea. This suggests that we should pay more attention to the intestinal mucosal microbiota in the mechanism study, the diagnostic biomarkers, and the drug development of mental stress diarrhea.

The animal model used in this experiment can better reflect the characteristics of mental stress-related diarrhea. The results of our previous studies (data integration reanalysis) showed that the comprehensive interventions led to growth retardation, lower thymus index and white blood cell count, and higher mean corpuscular hemoglobin concentration (Supplementary Figures S3A–E). We also observed that the intestinal propulsion rate was significantly increased in repeated stress-related diarrhea mice, while the comprehensive interventions led to a low expression of D-xylose in diarrhea and repeated stress mice, compared with control mice (Supplementary Figure S3F). It has been suggested that comprehensive interventions have caused extensive intestinal inflammation and malabsorption. Moreover, in this study, serum 5-HT was significantly decreased in the model mice, and it is an indicator of intestinal microbiota participating in the brain–gut nerve response to stress-related diarrhea (Ma et al., 2020; Spencer and Hu, 2020; Ming et al., 2021).

We found that in this study the alpha diversity of intestinal mucosal microbiota was significantly increased in repeated stress-related diarrhea mice, but the microbiota of intestinal contents showed only an upward trend, which indicated that the alpha diversity was unchanged in the microbiota of intestinal contents. In fact, intestinal bacterial overgrowth is often observed in patients with functional gastrointestinal disease (Shah et al., 2020). Other experimental studies have found that the diversity of intestinal microbiota is reduced in mice with functional stress-related diarrhea diseases such as irritable bowel syndrome (Konturek et al., 2020; Sugiyama and Shiotani, 2020), and such differences in the results may be related to the means and time of modeling interventions. The focus of this study is on the sensitivity of intestinal microbiota to changes in repeated stress-related diarrhea, so the corresponding modeling cycle was set. Meanwhile, according to our predictive function of microbiota, especially the expression of transcription genes related to microbiota proliferation in intestinal mucosal being significantly reduced, we speculate that the diversity of intestinal microbiota will decrease after the balance of microbiota is thoroughly disturbed with the prolongation of intervention time. Therefore, the effect of timing of functional stress-related diarrhea on intestinal microbiota deserves further study.

In this study, microbiotal OTU-based beta diversity analysis found no difference between groups. However, at the taxonomic species level, cluster analysis found that repeated stress-related diarrhea altered the species composition of microbiota both in intestinal contents and intestinal mucosa. Therefore, the subsequent analysis in this study focused on the differences at the taxonomic species level based on 16S rRNA PacBio SMRT gene full-length sequencing. It was found that the key bacterial species associated with mental diarrhea were mainly Proteobacteria. Up to now, some studies have demonstrated that Proteobacteria can reflect microbial dysbiosis or unstable intestinal microbial community structure (Shin et al., 2015). Proteobacteria expansion is a characteristic of intestinal epithelial dysregulation (Litvak et al., 2017); so, in this study, the key species of intestinal mucosal microbiota affected by repeated stress-related diarrhea mainly belong to Proteobacteria. So, intestinal epithelial disorder may occur in diarrhea mice in this study. Intestinal epithelial disorder is one of the pathological features of functional bowel disease including stress-related diarrhea (Grondin et al., 2020; Kim et al., 2020). The overall trend of microbiota community structure is an increase in pathogenic bacteria and a decrease in beneficial bacteria in both intestinal mucosa and intestinal contents. In the intestinal contents, the abundance of the pathogenic bacterium *Helicobacter ganmani*, a relative of *Helicobacter pylori* (Nagamine et al., 2015), was significantly increased. The results show an increase in the relative abundance of *Lactobacillus intestinalis* in the intestinal contents in repeated stress-related diarrhea mice, and it has been reported that *L. intestinalis* can aggravate the emotional disorder of diarrhea mice *via* the vagus nerve (Wang S. etal., 2020), which indicates that the imbalance of microbiota in this study may aggravate the psychological stress of repeated stress-related diarrhea mice. Moreover, the pathogen *Bacteroides vulgatus* has been reported to inhibit intestinal mucus secretion, leading to the development of Crohn’s disease, with a significant increase in abundance (Ramanan et al., 2014; Cuív et al., 2017). Especially in the intestinal mucosal microbiota, the abundance of pathogenic bacteria such as *Klebsiella pneumoniae* (Zhan et al., 2017) and *Escherichia* sp. BBDP27 increased significantly, while the abundance of the beneficial bacteria *Stigmatella aurantiaca* (Muller et al., 2019) decreased significantly. Notably, our study found that repeated stress diarrhea reduced the abundance of bacteria associated with mucus secretion (such as *S. aurantiaca*, a species of *Myxobacteria*) (Wolgemuth et al., 2002), while the abundance of bacteria with inhibitory effect on intestinal mucus secretion (such as *B. vulgatus*) increased significantly (Cuív et al., 2017). Recently, more and more attention has been paid to the regulation of the mucus barrier (Birchenough and Johansson, 2020). As recognized in a recent review, intestinal mucus is part of innate intestinal mucosal barrier, which is involved in reducing the exposure of antigens and bacteria to the immune system under intestinal epithelial cells (Paone and Cani, 2020). Therefore, the disorder of bacterial synthesis and metabolism mucus may be one of the key factors of diarrhea caused by repeated stress.

In this study, the analysis of microbiota metagenomic function found that repeated stress-related diarrhea can reduce the relative abundance of bacteria with the function of carbohydrate metabolism both in intestinal content and intestinal mucosa microbiota. Diarrhea is a metabolic disease, which mainly causes digestive disorders. The process of digestion is catalyzed by enzymes that are either endogenous or produced by the host’s microbial population (Janiak, 2016). Diarrhea reduces the digestive abilities of the microbial community present in the host’s intestines, and the decrease of digestive enzyme activity of intestinal microbiota causes or aggravates diarrhea. Intestinal microbiota is an important part of the host’s digestive system, and the digestive function of intestinal microbiota is related to dyspepsia caused by diarrhea. The digestive enzyme activity of the intestine can explain the digestive function of intestinal microbiota to a certain extent. Therefore, we further determined the digestive enzyme activities related to microbial carbohydrate metabolism including protease, lactase, amylase, invertase, cellulase, and xylanase in intestinal content and mucosal samples (data integration reanalysis). We observed that in normal mice, the activity of microbial lactase in intestinal contents was significantly higher than that in intestinal mucosa, while the microbial xylanase activity increased significantly in intestinal mucosa. Amylase activity was significantly reduced in both intestinal contents and mucosa, while repeated stress-related diarrhea led to the activity of protease, invertase, and xylanase being decreased significantly only in the intestinal mucosa but not in the intestinal contents (Supplementary Figure S4). This indicates that the activity of microbial enzymes is more sensitive in response to repeated stress-related diarrhea in the intestinal mucosa. This is consistent with the result of microbiota metagenomic functional prediction in this study.

Moreover, we predicted the function of bacterial genes and found that there were disorders of amino acid metabolism, including the amino acid nitrogen fixation pathway, caused by microbiota in the intestinal mucosa. The results showed that bacterial genes mainly induced intestinal inflammation through the FoxO signaling pathway (Douglas et al., 2015) and MAPK signaling pathway (Grondin et al., 2020) and participated in neural response. It is suggested that the metabolic activities of intestinal microbiota are involved in the brain–gut nerve response, including tryptophan metabolism. 5-HT is a biological derivative of tryptophan, which is mainly synthesized by bacteria. It is an important amino acid substance of the brain–gut nerve interaction (Ma et al., 2020). Therefore, we established models of intestinal content and intestinal mucosal microbiota by LDA machine learning and analyzed the correlation between the two microbiotal community systems and serum 5-HT. The correlation further confirmed that the function of intestinal mucosal microbiota is linked more to repeated stress-related diarrhea compared with intestinal contents.

In conclusion, the present study provides novel insight into the biodiversity, taxonomic bacteria rank composition, and microbiota potential function in two gut regions (including intestinal contents and intestinal mucosa), which respond to repeated stress-related diarrhea. Compared with intestinal content microbiota, the diversity and composition of microbiota colonized in intestinal mucosa are more sensitive to repeated stress-related diarrhea. Repeated stress-related diarrhea is more likely to perturb the microbiota predictive function in the intestinal mucosa. Moreover, the intestinal mucosa microbiota might serve as a novel potential prediction model for serum 5-HT concentration involvement in repeated stress-related diarrhea.

## Data Availability Statement

The datasets presented in this study can be found in online repositories. The names of the repository/repositories and accession number(s) can be found below: NCBI (accession: PRJNA679717).

## Ethics Statement

The animal study was reviewed and approved by the Animal Ethics and Welfare Committee of Hunan University of Chinese Medicine.

## Author Contributions

Z-JT designed the study. C-YZ wrote the manuscript. C-YZ and YW analyzed the data. H-QS and X-XP performed the experiments. X-YL checked the manuscript. The decision to submit the manuscript for publication was made by all the authors. All authors contributed to the article and approved the submitted version.

## Conflict of Interest

The authors declare that the research was conducted in the absence of any commercial or financial relationships that could be construed as a potential conflict of interest.

## Footnotes

1 http://ehbio.com/Cloud_Platform/front/#/

2 http://login.majorbio.com/login

3 http://huttenhower.sph.harvard.edu/galaxy/

4 https://github.com/picrust/picrust2

5 http://echarts.baidu.com/download.html

## Supplementary Material

Supplementary Figure 1 16S rRNA Pacbio SMRT gene full-length sequencing results of intestinal contents and mucosal microbiota in repeated stress-related diarrhea and normal control mice. **(A)** Sequence length distribution; the abscissa is the length distribution of all samples, while the ordinate is the total number of sequences corresponding to each length. **(B)** Species accumulation curves; the abscissa represents the sample size, the ordinate represents the number of detected species, and the blue shadow reflects the confidence interval of the curve; the curve tends to be gentle, indicating that the sample size is enough to reflect the richness of the community. **(C)** Rarefaction curve predicts the total number of species and the relative abundance of each species in a given sequence of sequencing depths. **(D)** Number of common and unique OTUs in each group. Cc, intestinal contents in control mice; Cds, intestinal contents in repeated stress-related diarrhea mice; Mc, intestinal mucosa in control mice; Mds, intestinal mucosa in repeated stress-related diarrhea mice.

Supplementary Figure 2 Cluster analysis of PCoA1 and NMDS of OTUs (Bray Curtis and Unweighted UniFrac). **(A)** PCoA1. **(B)** NMDS. Cc, intestinal contents in control mice; Cds, intestinal contents in repeated stress-related diarrhea mice; Mc, intestinal mucosa in control mice; Mds, intestinal mucosa in repeated stress-related diarrhea mice.

Supplementary Figure 3 The characteristics in the repeated stress-related diarrhea based on the data integration reanalysis. **(A)** Body weight; **(B)** Intestinal propulsion rate; **(C)** Thymus index; **(D)** Mean corpuscular hemoglobin concentration; **(E)** Weight cell count; **(F)** D-xylose. ^∗^*p* < 0.05, ^∗∗^*p* < 0.01, ^∗∗∗^ *p* < 0.001, N.S.: not significant (*p* > 0.05). Cc, intestinal contents in control mice; Cds, intestinal contents in repeated stress-related diarrhea mice; Mc, intestinal mucosa in control mice; Mds, intestinal mucosa in repeated stress-related diarrhea mice.

Supplementary Figure 4 Activity of microbial enzymes in the repeated stress-related diarrhea in the intestinal mucosa. ^∗^*p* < 0.05, ^∗∗^*p* < 0.01, ^∗∗∗^ *p* < 0.001, N.S.: not significant (*p* > 0.05). Cc, intestinal contents in control mice; Cds, intestinal contents in repeated stress-related diarrhea mice; Mc, intestinal mucosa in control mice; Mds, intestinal mucosa in repeated stress-related diarrhea mice.
