# Supplementary material for: Gut Microbiota Comparison Between Intestinal Contents and Mucosa in Mice With Repeated Stress-Related Diarrhea Provides Novel Insight
Source: Front Microbiol. 2021 Feb 23;12:626691. doi: 10.3389/fmicb.2021.626691 (PMC7940357; doi:10.3389/fmicb.2021.626691)
Supplement: Supplementary Figure 1 — 16S rRNA Pacbio SMRT gene full-length sequencing results of intestinal contents and mucosal microbiota in repeated stress-related diarrhea and normal control mice. (A) Sequence length distribution; the abscissa is the length distribution of all samples, while the ordinate is the total number of sequences corresponding to each length. (B) Species accumulation curves; the abscissa represents the sample size, the ordinate represents the number of detected species, and the blue shadow reflects the confidence interval of the curve; the curve tends to be gentle, indicating that the sample size is enough to reflect the richness of the community. (C) Rarefaction curve predicts the total number of species and the relative abundance of each species in a given sequence of sequencing depths. (D) Number of common and unique OTUs in each group. Cc, intestinal contents in control mice; Cds, intestinal contents in repeated stress-related diarrhea mice; Mc, intestinal mucosa in control mice; Mds, intestinal mucosa in repeated stress-related diarrhea mice. [file Data_Sheet_1.pdf]

Supplementary Material

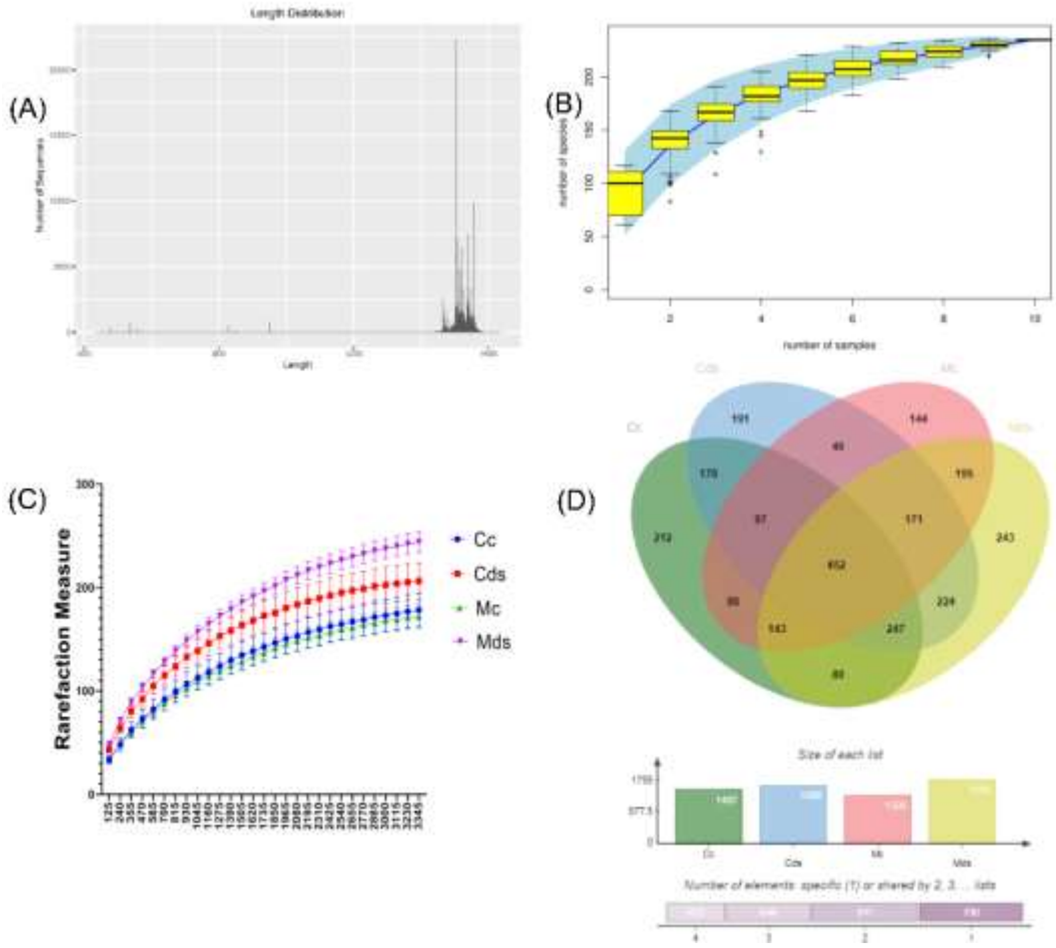

Supplementary figure 1. 16S rRNA Pacbio SMRT gene full-length sequencing results of intestinal contents and mucosal microbiota in repeated stress-related diarrhoea and normal control mice. A, Sequence length distribution; the abscissa is the length distribution of all samples, while the ordinate is the total number of sequences corresponding to each length. **B**, Species accumulation curves; the abscissa represents the sample size, the ordinate represents the number of detected species, and the blue shadow reflects the confidence interval of the curve; the curve tends to be gentle, indicating that the sample size is enough to reflect the richness of the community. **C**, Rarefaction curve predicts the total number of species and the relative abundance of each species in a given sequence of sequencing depths. **D**, Number of common and unique OTUs in each group.

Cc, intestinal contents in control mice; Cds, intestinal contents in repeated stress-related diarrhoea mice; Mc, intestinal mucosa in control mice; Mds, intestinal mucosa in repeated stress-related diarrhoea mice.

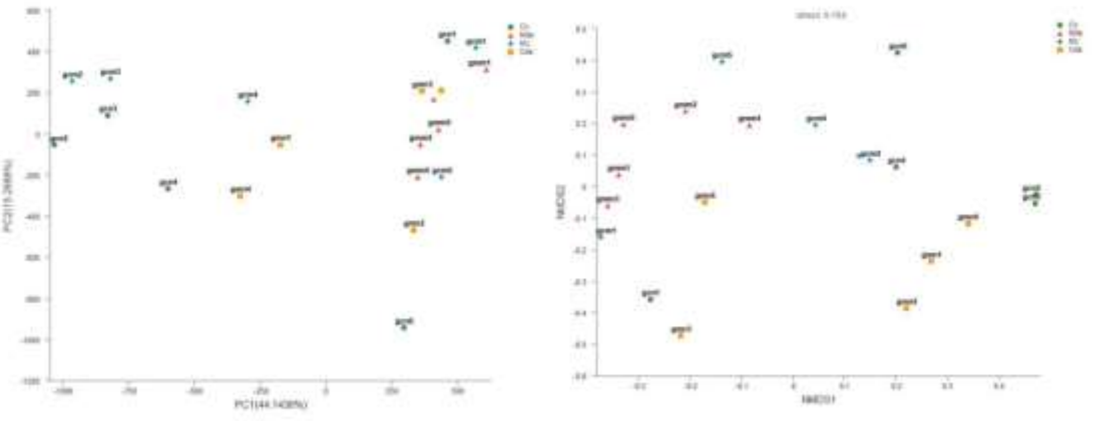

(A) (B)

Supplementary figure 2. Cluster analysis of PCoA1 and NMDS of OTUs (Bray Curtis and Unweighted UniFrac). **A**, PCoA1. **B**, NMDS.

Cc, intestinal contents in control mice; Cds, intestinal contents in repeated stress-related diarrhoea mice; Mc, intestinal mucosa in control mice; Mds, intestinal mucosa in repeated stress-related diarrhoea mice.

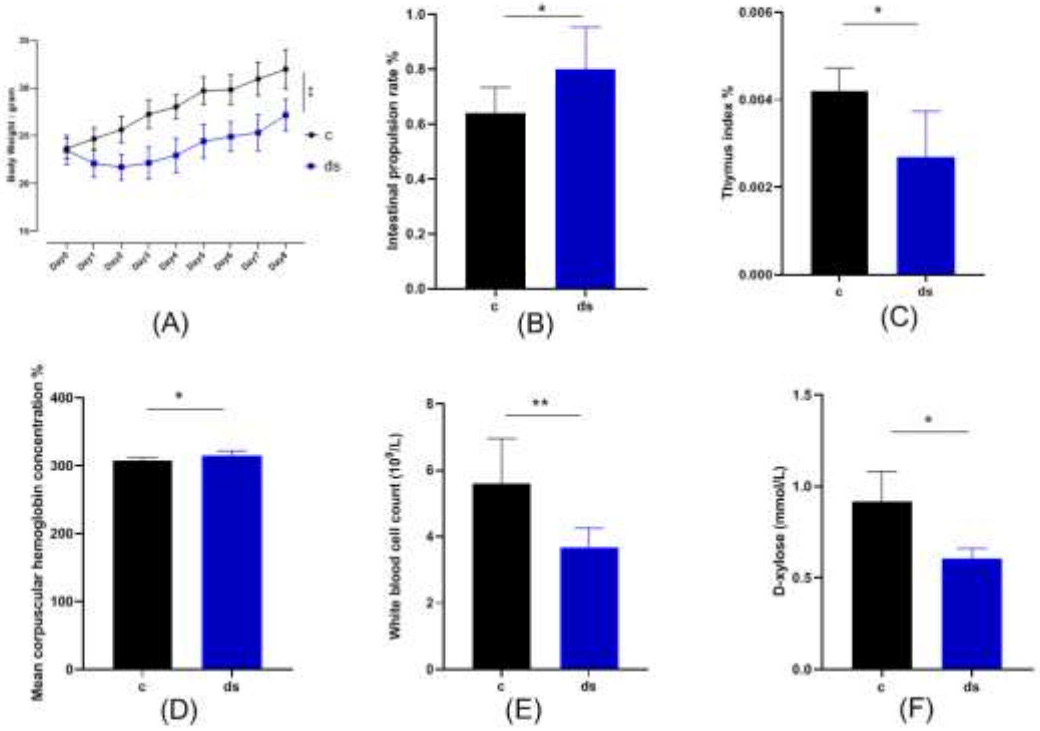

Supplementary figure 3. The characteristics in the repeated stress-related diarrhoea based on the data integration reanalysis. **A**, Body weight; **B**, Intestinal propulsion rate; **C**, Thymus index; **D**, Mean corpuscular hemoglobin concentration; **E**, Weight cell count; **F**, D-xylose.

*$p < 0.05$, **$p < 0.01$, *** $p < 0.001$, N.S.: not significant ($p > 0.05$).

Cc, intestinal contents in control mice; Cds, intestinal contents in repeated stress-related diarrhoea mice; Mc, intestinal mucosa in control mice; Mds, intestinal mucosa in repeated stress-related diarrhoea mice.

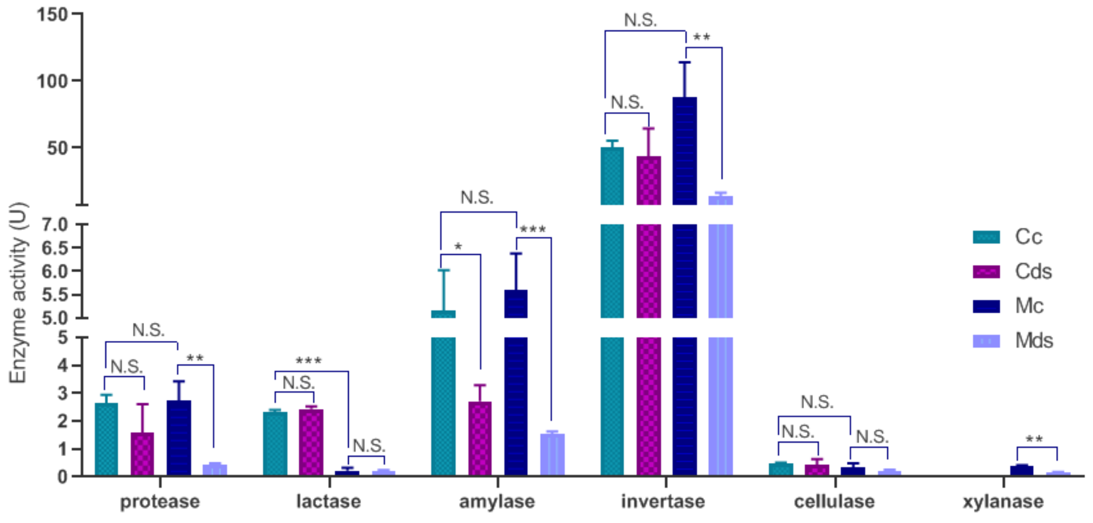

Supplementary figure 4. Activity of microbial enzymes in the repeated stress-related diarrhoea in the intestinal mucosa. *$p < 0.05$, **$p < 0.01$, *** $p < 0.001$, N.S.: not significant ($p > 0.05$).

Cc, intestinal contents in control mice; Cds, intestinal contents in repeated stress-related diarrhoea mice; Mc, intestinal mucosa in control mice; Mds, intestinal mucosa in repeated stress-related diarrhoea mice.
